# Electrical Restitution and Its Modifications by Antiarrhythmic Drugs in Undiseased Human Ventricular Muscle

**DOI:** 10.3389/fphar.2020.00479

**Published:** 2020-04-30

**Authors:** Tamás Árpádffy-Lovas, István Baczkó, Beáta Baláti, Miklós Bitay, Norbert Jost, Csaba Lengyel, Norbert Nagy, János Takács, András Varró, László Virág

**Affiliations:** ^1^Department of Pharmacology and Pharmacotherapy, Faculty of Medicine, University of Szeged, Szeged, Hungary; ^2^Department of Pharmacology and Pharmacotherapy, Interdisciplinary Excellence Centre, University of Szeged, Szeged, Hungary; ^3^Department of Cardiac Surgery, Faculty of Medicine, University of Szeged, Szeged, Hungary; ^4^MTA-SZTE Research Group for Cardiovascular Pharmacology, Hungarian Academy of Sciences, Szeged, Hungary; ^5^First Department of Internal Medicine, University of Szeged, Szeged, Hungary

**Keywords:** arrhythmia, action potential, electrical restitution, human ventricle, cardiac electrophysiogy

## Abstract

**Introduction:**

Re-entry is a basic mechanism of ventricular fibrillation, which can be elicited by extrasystolic activity, but the timing of an extrasystole can be critical. The action potential duration (APD) of an extrasystole depends on the proximity of the preceding beat, and the relation between its timing and its APD is called electrical restitution. The aim of the present work was to study and compare the effect of several antiarrhythmic drugs on restitution in preparations from undiseased human ventricular muscle, and other mammalian species.

**Methods:**

Action potentials were recorded in preparations obtained from rat, guinea pig, rabbit, and dog hearts; and from undiseased human donor hearts using the conventional microelectrode technique. Preparations were stimulated with different basic cycle lengths (BCLs) ranging from 300 to 5,000 ms. To study restitution, single test pulses were applied at every 20th beat while the preparation was driven at 1,000 ms BCL.

**Results:**

Marked differences were found between the animal and human preparations regarding restitution and steady-state frequency dependent curves. In human ventricular muscle, restitution kinetics were slower in preparations with large phase 1 repolarization with shorter APDs at 1000 ms BCL compared to preparations with small phase 1. Preparations having APD longer than 300 ms at 1000 ms BCL had slower restitution kinetics than those having APD shorter than 250 ms. The selective I_Kr_ inhibitors E-4031 and sotalol increased overall APD and slowed the restitution kinetics, while I_Ks_ inhibition did not influence APD and electrical restitution. Mexiletine and nisoldipine shortened APD, but only mexiletine slowed restitution kinetics.

**Discussion:**

Frequency dependent APD changes, including electrical restitution, were partly determined by the APD at the BCL. Small phase 1 associated with slower restitution suggests a role of I_to_ in restitution. APD prolonging drugs slowed restitution, while mexiletine, a known inhibitor of I_Na_, shortened basic APD but also slowed restitution. These results indicate that although basic APD has an important role in restitution, other transmembrane currents, such as I_Na_ or I_to_, can also affect restitution kinetics. This raises the possibility that ion channel modifier drugs slowing restitution kinetics may have antiarrhythmic properties by altering restitution.

## Introduction

Cardiovascular diseases are the leading causes of mortality in Western countries including the USA, Germany, France, and the UK. In approximately 50% of the cases the cause of death in cardiac patients is sudden cardiac death due to ventricular fibrillation (Jazayeri and Emert, 2019). The underlying mechanisms of ventricular fibrillation are complex, often multi-factorial, and are still not fully understood, therefore, they are subjects of current investigations. In general, arrhythmias can be explained by impaired impulse conduction and/or abnormal automaticity within the heart. The cellular cause of impulse conduction defects can have a distinct anatomical cause exhibiting a fixed pathway, usually determined by ischemic or fibrotic injury (Nguyen et al., 2017; Himel et al., 2019). Alternatively, the re-entry pathway can form without such injuries, due to enhanced dispersion of repolarization and consequently enhanced dispersion of refractoriness (Himel et al., 2019). The latter determines the ability of the ventricular muscle to be re-excited following a previous beat. In case the differences in action potential duration (APD), and consequently the effective refractory period (ERP) are enhanced, i.e. dispersion of APD or repolarization is augmented, the propagation of an early extra beat can be delayed or blocked in the direction that has myocytes with longer APDs, but conducted normally to the direction that has myocytes with shorter APDs. Therefore, in such an area, the extra beat can travel in a zig-zag pattern and can re-enter into areas that have been previously excited, eliciting chaotic rhythm or even fibrillation. Accordingly, the timing of an extrasystole is critical for arrhythmogenesis (Akar et al., 2002; Tran et al., 2007; Zaniboni, 2019). It has been known for a long time that the APD/ERP of an extrasystole depends on the proximity of the preceding beat, called diastolic interval; and as the diastolic intervals increase, the APDs/ERPs of the extra beats also increase. This process is called electrical restitution and had been described long ago (Nolasco and Dahlen, 1968; Boyett and Jewell, 1978; Elharrar and Surawicz, 1983), but its importance in arrhythmia research gained particular attention again in the past two decades (Gilmour, 2002; Franz, 2003; Kalb et al., 2004; Gilmour, 2009; Orini et al., 2016; Osadchii, 2017a; Osadchii, 2017b; Shattock et al., 2017; Orini et al., 2019; Osadchii, 2019). According to the restitution hypothesis, as diastolic intervals increase due to propagation of an extrasystole, the next following possible extrasystole would encounter prolonged APD/ERP and local conduction defect can occur. A steeper or faster restitution curve would favor such an effect and would be considered proarrhythmic; flattened or slower electrical restitution would have the opposite effect (Garfinkel et al., 2000; Qu et al., 2014; Shattock et al., 2017; Osadchii, 2017a; Osadchii, 2017b). Several studies in different preparations investigated the effects of antiarrhythmic drugs on the cardiac electric restitution properties (Varró et al., 1985; Hsieh et al., 2013; Osadchii, 2017a; Osadchii, 2017b; Shattock et al., 2017). These studies yielded different results depending on the protocols (dynamic or standard), on the basic stimulation frequencies, on the preparations (ventricular muscle or Purkinje fibers), and on the species (guinea-pig, rabbit, rat, or dog) used in their experimental approaches (Elharrar and Surawicz, 1983; Kalb et al., 2004; Orini et al., 2016; Shattock et al., 2017; Osadchii, 2019). The species used may have special significance, since, as **Figure 2** shows, there are marked differences between restitution curves measured in ventricular papillary muscle from different species (in rat, guinea-pig, rabbit, dog or human preparations) with the same experimental restitution pacing protocol and basic stimulation frequency. Therefore, the aim of the present work was to study the effect of several antiarrhythmic drugs on undiseased human ventricular muscle to better understand the possible implications of drug effects on electrical restitution, and understand these effects in human arrhythmogenesis.

## Methods

### Human General Donor Cardiac Tissue Ethics Statement

Hearts were obtained from general organ donors whose undiseased hearts were explanted to obtain pulmonary and aortic valves for transplant surgery. Before cardiac explantation, organ donors did not receive medication apart from dobutamine, furosemide, and plasma expanders. According to the Hungarian law to obtain samples from donors, the consent of the patients or relatives is not needed. Therefore, consent is waived under local legislation. The investigations conformed to the principles of the Declaration of Helsinki. Experimental protocols were approved by the National Scientific and Research Ethical Review Boards (4991-0/2010-1018EKU [339/PI/010]).

### Animals

All experiments were carried out in compliance with the Guide for the Care and Use of Laboratory Animals (USA NIH publication NO 85-23, revised 1996) and conformed to the Directive 2010/63/EU of the European Parliament. The protocols have been approved by the Ethical Committee for the Protection of Animals in Research of the University of Szeged, Szeged, Hungary (approval number: I-74-24-2017) and by the Department of Animal Health and Food Control of the Ministry of Agriculture and Rural Development (authority approval number XIII/3331/2017).

### Conventional Microelectrode Technique

Action potentials were recorded in right ventricular trabeculae or papillary muscle preparations obtained from rat, guinea pig, rabbit, dog hearts, and from undiseased human donor hearts using the conventional microelectrode technique.

Rats (either sex, 200–400 g), guinea-pigs (either sex, 400–600 g), rabbits (either sex, 2.5–3.5 kg) and dogs (either sex, 10–15 kg) were anesthetized by sodium pentobarbitone (30 mg/kg i.p. for rat and guinea pig, i.v. for rabbit and dog) following sedation (xylazine 1 mg/kg). The animals also received intravenous injection of 400 U/kg heparin. In case of human donor hearts, immediately after explantation, each heart was perfused with cardioplegic solution and kept cold (4–6°C) for 2–4 hours before dissection.

Preparations were individually mounted in a tissue chamber with a volume of 50 ml. During experiments, modified Locke's solution was used, containing (in mM): NaCl, 128.3; KCl, 4; CaCl_2_, 1.8; MgCl_2_, 0.42; NaHCO_3_, 21.4; and glucose, 10. The pH of this solution was set between 7.35 and 7.4 when gassed with the mixture of 95% O_2_ and 5% CO_2_ at 37°C. Each preparation was stimulated through a pair of platinum electrodes in contact with the preparation using rectangular current pulses of 1 to 3 ms duration at twice of the threshold strength at a constant basic cycle length of 1000 ms (S1). These stimuli were delivered for at least 60 min allowing the preparation to equilibrate before the measurements were initiated. Transmembrane potentials were recorded using conventional glass microelectrodes, filled with 3 M KCl, and having tip resistances of 5–20 MΩ, connected to the input of a high impedance electrometer (Experimetria, type 309, Budapest, Hungary) which was coupled to a dual beam oscilloscope.

The resting potential (RP), action potential amplitude (APA), maximum upstroke velocity (V_max_), and APD measured at 50% and 90% of repolarization (APD_50_ and APD_90_, respectively) were determined off-line using an in-house developed software (APES) running on a computer equipped with an ADA 3300 analog-to-digital data acquisition board (Real Time Devices, Inc., State College, Pennsylvania) having a maximum sampling frequency of 40 kHz.

The following types of stimulations were applied in the course of the experiments: stimulation with a constant cycle length of 1000 ms; stimulation with different constant cycle lengths ranging from 300 to 5000 ms. To determine the recovery kinetics of APD_90_ (APD_90_ restitution), extra test action potentials were elicited by using single test pulses (S2) in a preparation driven at a basic cycle length of 1000 ms. The S1–S2 coupling interval was increased progressively from the end of the refractory period. The effective refractory period was defined as the longest S1–S2 interval at which S2 failed to elicit a propagated response. The diastolic intervals preceding the test action potential were measured from the point corresponding to 90% of repolarization of the preceding basic beat to the upstroke of the test action potential and were increased progressively.

Attempts were made to maintain the same impalement throughout each experiment. In case an impalement became dislodged, adjustment was attempted, and if the action potential characteristics of the re-established impalement deviated by less than 5% from the previous measurement, the experiment continued. All measurements were performed at 37°C.

### Data Analysis

All data are expressed as means ± SEM. The “n” number refers to the number of experiments (i.e. the number of ventricular muscle preparations). Data points of restitution curves were fitted by a mono-exponential function in order to calculate the kinetic time constant of the APD_90_ restitution process:

$$\text{APD}={\text{APD}}_{\text{ss}}-\text{A}*\exp(-DI/\tau )$$

where APD_ss_ is the maximal action potential duration (APD_90_), A is the amplitude of the exponential function, DI is the diastolic interval, and τ is the time constant.

## Results

**Figure 1:**
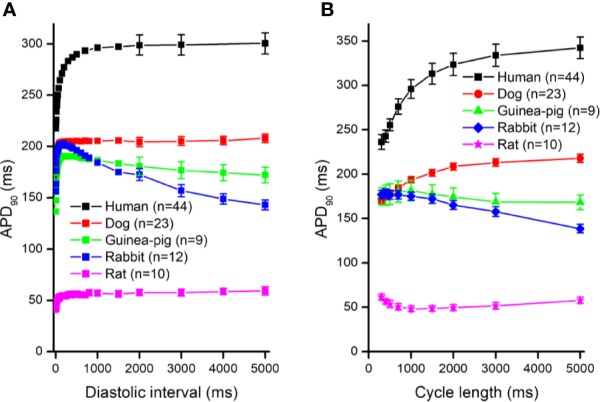
Action potential duration (APD_90_) restitution curves (panel **A**) and the steady-state cycle length dependence of the action potential duration (panel **B**) in human, dog, guinea pig, rabbit, and rat right ventricular muscle preparations. For the sake of clarity, the SEM values were indicated in case of diastolic intervals 2000 – 5000 ms in (panel **A**).

**Figure 2:**
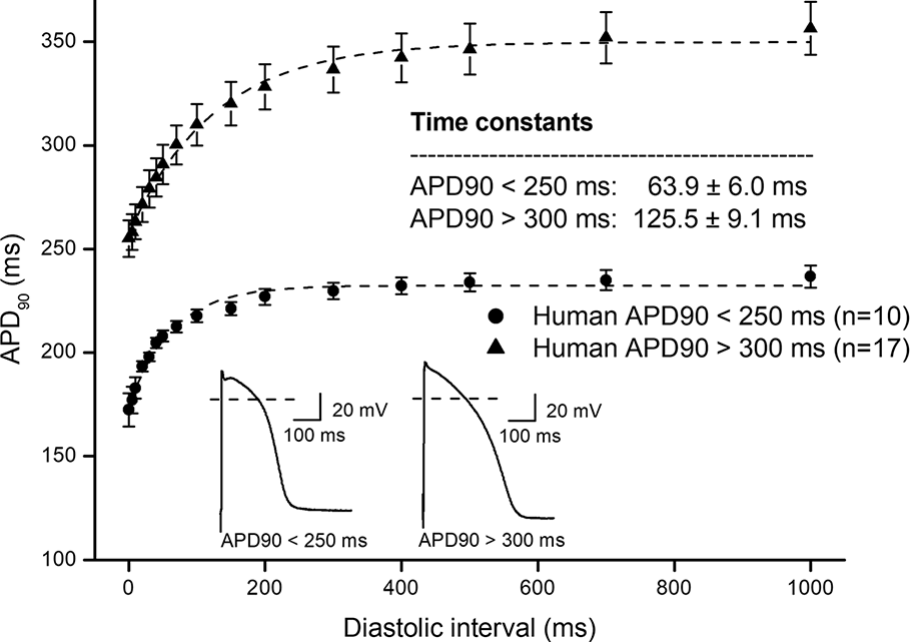
Comparing human ventricular electrical restitution curves based on the action potential duration. Human APD_90_ restitution curves were separated into short APD (APD_90_ < 250 ms) and long APD (APD_90_ > 300 ms) groups. The data points up to 1000 ms diastolic interval were fitted by single exponential function. The inset shows the kinetical time constants for the two groups.

**Figure 3:**
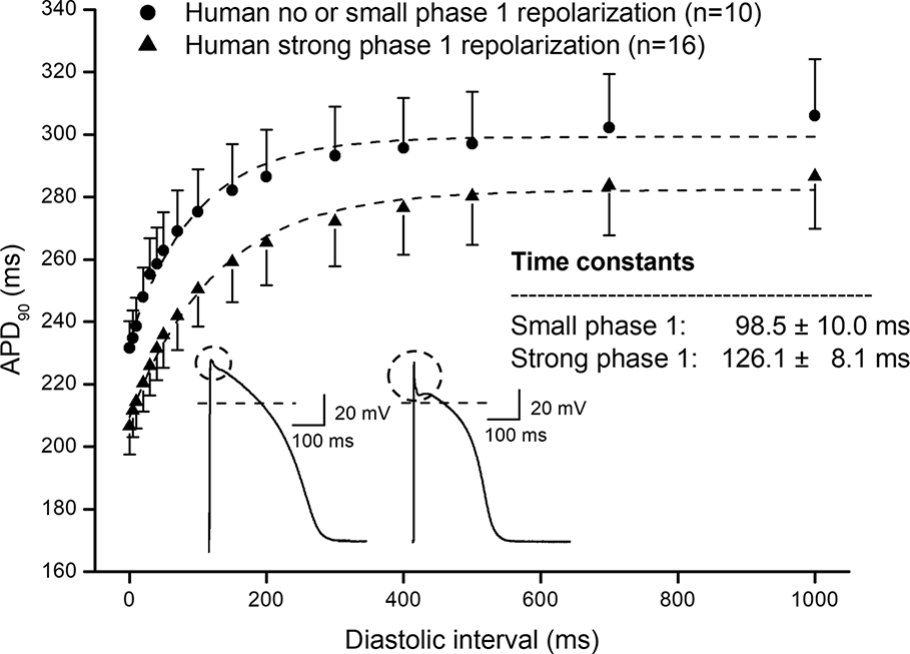
Comparing human ventricular action potential duration restitution curves based on the amplitude of phase 1 repolarization. Human APD_90_ restitution curves were separated into two groups, one showed prominent phase 1 repolarization and another one had no or small phase 1 repolarization. The data points up to 1000 ms diastolic interval were fitted by single exponential function. The inset shows the kinetical time constants for the two groups.

In **Figure 1**, frequency dependent APD changes are shown in different species including human following various constant steady-state (S1–S1) and abrupt changes of cycle lengths (S1–S2). The figure shows that there are marked differences both in the electrical restitution and steady-state frequency dependent curves. The nature and mechanism of the frequency dependent APD changes (Carmeliet, 1977; Elharrar and Surawicz, 1983; Obreztchikova et al., 2006; Qu et al., 2014; Ni et al., 2019) including electrical restitution are not fully resolved yet. A recent study of Schattock et al. (2017) suggested that the slope of the restitution curve depends on the APD of the basic heart rate. Therefore, as **Figure 2** shows, human ventricular electrical restitution curves separated according to their action potential durations at basic cycle length of 1000 ms. In preparations with APD_90_ shorter than 250 ms, the time constant (τ) was 63.9 ± 6.0 ms (n = 10), while in preparations with APD_90_ longer than 300 ms τ was 125.5 ± 9.1 ms (n = 17). **Figure 2** indicates that electrical restitution kinetics are slower as action potential durations increase, suggesting that restitution kinetics, at least partly, indeed depend on intrinsic behavior of the repolarization process. In addition, as shown in **Figure 3**, human ventricular APD restitution curves have somewhat slower restitution kinetics where the basic action potentials showed prominent phase 1 repolarization during the plateau phase (τ = 126.1 ± 8.1 ms, n = 16) compared to those that had no strong phase 1 repolarization (τ = 98.5 ± 10.0 ms, n = 10), suggesting a possible role of I_to_ in the restitution process. In this respect, it is worth to note that APDs in preparations having prominent phase 1 repolarization were shorter than those having no or small phase 1 repolarization. Also, rabbit restitution curves and steady-state frequency-dependent APD have a declining slope at diastolic intervals and cycle lengths longer than 1000 ms. Since in rabbit I_to_ is characterized by slow recovery (Fermini et al., 1992; Sánchez-Chapula et al., 1994), these results also suggest a possible role of I_to_ in the cycle length dependent APD changes including restitution.

**Figure 4:**
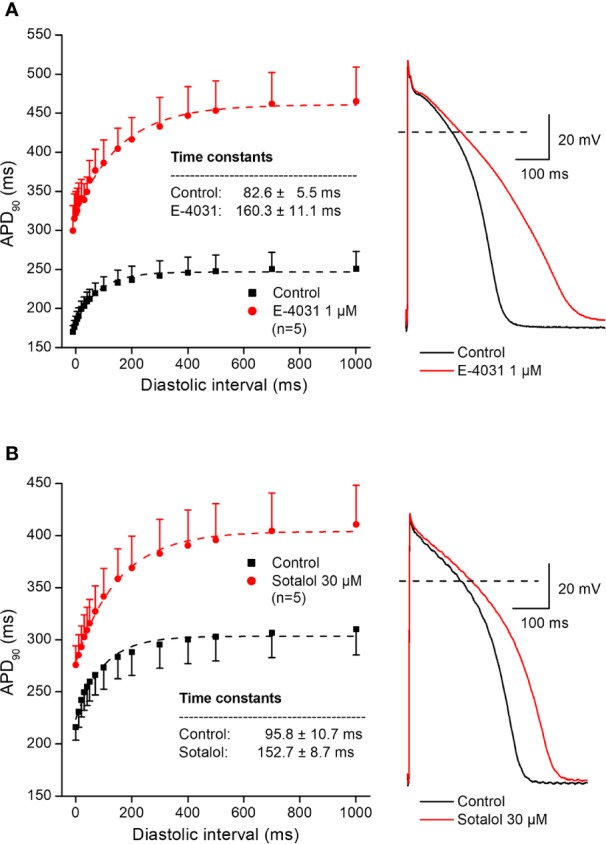
Effect of two selective rapid delayed rectifier inhibitor antiarrhythmic drugs – E-4031 (panel **A**) and sotalol (panel **B**) – on the human electrical restitution curve. The data points up to 1000 ms diastolic interval were fitted by single exponential function. The inset shows the kinetical time constants in control conditions and after drug application. On the right part of the figure original action potential traces are shown before and after drug application at basic cycle length of 1000 ms.

**Figure 5:**
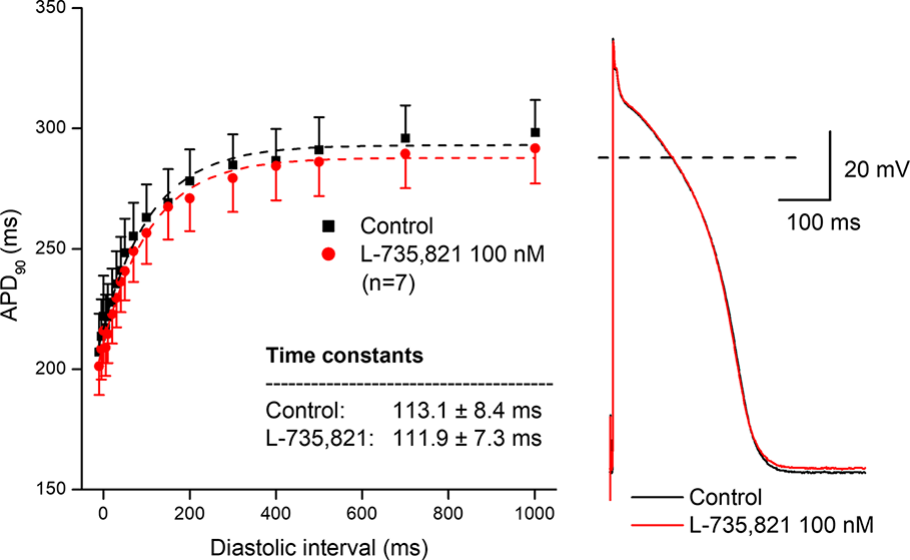
Lack of effect of the selective slow delayed rectifier inhibitor L-735,821 on the human electrical restitution curve. The data points up to 1000 ms diastolic interval were fitted by single exponential function. The inset shows the kinetical time constants in control conditions and after application of L-735,821. On the right part of the figure original action potential traces are shown before and after drug application at basic cycle length of 1000 ms.

**Figure 6:**
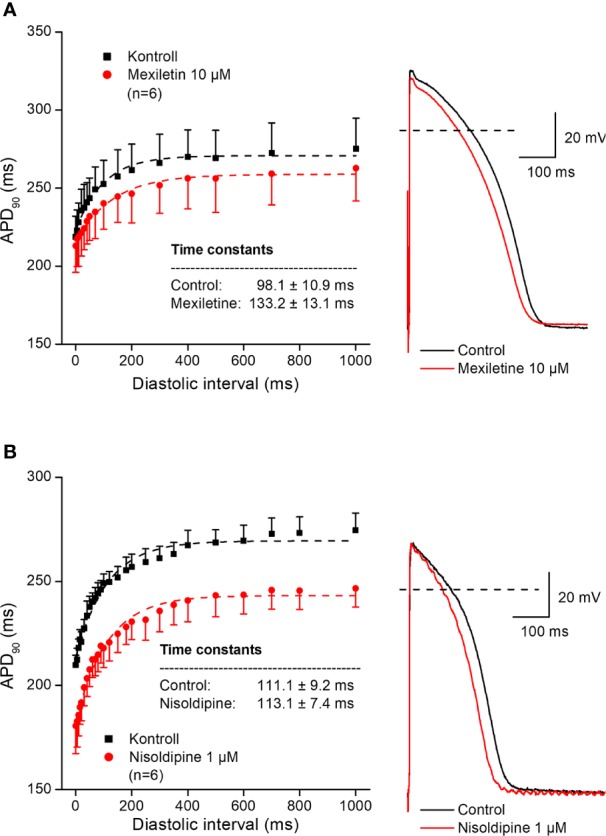
Effect of the sodium channel inhibitor mexiletine (panel **A**) and the L-type calcium current blocker nisoldipine (panel **B**) on the human electrical restitution curve. The data points up to 1000 ms diastolic interval were fitted by single exponential function. The inset shows the kinetical time constants in control conditions and after drug application. On the right part of the figure original action potential traces are shown before and after drug application at basic cycle length of 1000 ms.

In further experiments, the effects of several antiarrhythmic drugs were studied on the electrical restitution curves in human undiseased ventricular muscle preparations. **Figure 4** shows that the selective rapid delayed rectifier potassium current (I_Kr_) inhibitor E-4031 and sotalol increased overall APD and slowed the kinetics of the restitution curve (from τ = 82.6 ± 5.5 ms to τ = 160.3 ± 11.1 ms, n = 5; and from τ = 95.8 ± 10.7 ms to τ = 152.7 ± 8.7 ms, n = 5, respectively). **Figure 5** illustrates that L-735,821, a specific inhibitor of the slow delayed rectifier potassium current (I_Ks_) does not influence APD and electrical restitution curves (τ = 113.1 ± 8.4 ms vs. τ = 111.9 ± 7.3 ms, n = 7). In further experiments, the effects of the inward sodium current (I_Na_) inhibitor mexiletine and the inward L-type calcium current blocker nisoldipine were studied on human ventricular electrical restitution curves. **Figure 6** shows that both mexiletine and nisoldipine shortened APD but only mexiletine slowed restitution kinetics in human ventricular muscle preparations (from τ = 98.1 ± 10.9 ms to τ = 133.2 ± 13.1 ms, n = 6; from τ = 111.1 ± 9.2 ms to τ = 113.1 ± 7.4 ms, n = 6, respectively).

## Discussion

In this study, the electrical restitution of APD and its possible influence by several antiarrhythmic drugs in human ventricular muscle was investigated. Notwithstanding plentiful data in different animal experiments, according to our best knowledge, there is no systemic study on electrical restitution available in undiseased human ventricular muscle with the conventional microelectrode technique.

The main novel findings in the present work are as follows;

1. Human ventricular APD restitution differs from those reported in other species.
2. In spite of the marked species differences in the ventricular restitution curve, in human ventricle, similar to those reported earlier in other mammalian species (Shattock et al., 2017), longer repolarization is associated with slower restitution kinetics.
3. However, human ventricle exhibiting prominent phase 1 repolarization, presumably due to high level of I_to_ expression, was associated with shorter APD but slower restitution kinetics.
4. Drugs that inhibit I_Kr_ and I_Na_ slow restitution kinetics of APD restitution curve but drugs inhibiting I_Ks_ do not influence electrical APD restitution curves in human ventricular muscle.

APD restitution is an important process in the adaptation of the action potential to abrupt changes in cycle length and has been postulated playing an important role in the susceptibility to re-entrant arrhythmias, such as ventricular fibrillation (Garfinkel et al., 2000; Gilmour, 2002; Toal et al., 2009; Qu et al., 2014; Orini et al., 2016; Osadchii, 2017a; Osadchii, 2017b). Accordingly, it is generally agreed that slower restitution kinetics and a less steep restitution slope would result in antiarrhythmic effects, while steeper and faster restitution would be proarrhythmic (Garfinkel et al., 2000; Gilmour, 2002; Qu et al., 2014; Osadchii, 2017a; Osadchii, 2017b; Shattock et al., 2017; Zaniboni, 2019). As diastolic intervals are increasing due to propagation of an extra beat, a next short coupled extra beat would encounter longer APD or ERP and, as a result of this, local conduction block can develop. A steeper restitution curve would facilitate this possibility with potential proarrhythmic consequences, but a flattened restitution curve would have the opposite effect.

Repolarization of cardiac ventricular muscle has been known for long to be dependent on species and stimulation frequency (Carmeliet, 1977; Boyett and Jewell, 1978). The cellular and subcellular mechanisms of APD restitution have been studied extensively. However, they are still subjects of debate (Boyett and Jewell, 1978; Elharrar and Surawicz, 1983; Hsieh et al., 2013; Osadchii, 2017a; Osadchii, 2017b; Shattock et al., 2017; Zaniboni, 2019). Frequency dependent APD changes including APD restitution in case the cycle length or diastolic interval ranges are sufficiently long can be characterized by multiple exponential fits (Elharrar and Surawicz, 1983; Varró et al., 1985). The rapid exponential components of these fits are generally attributed to deactivation and recovery from inactivation properties of various ion channels activated during the previous baseline beats, as well as intracellular and extracellular ion concentration changes, which directly or indirectly alter electrogenic pumps and exchangers, often called collectively as “short term memory” (Elharrar and Surawicz, 1983; Toal et al., 2009). Changes in the expression of ion channels can cause the so-called long-term memory (Obreztchikova et al., 2006), which was not investigated in our experiments.

In a recent study by Shattock et al. (2017) it was suggested that APD restitution kinetics were determined by the length of the APD of the basic beat. This speculation was based on guinea pig and rabbit experiments in Langendorff preparation measuring monophasic APD with a Franz catheter, or with the sharp microelectrode technique in single isolated guinea-pig myocytes applying the dynamic restitution protocol (Shattock et al., 2017). In this study, a wide range of drugs that all prolong APD by different modes of actions (such as clofilium, Bay K 8644, veratridine, catecholamines; and interventions such as low extracellular Ca^2+^ and transverse aortic constriction induced heart failure) slowed the kinetics or flattened the restitution curves. Based on these results, in agreement with the hypothesis of Zaza (Zaza and Varró, 2006; Zaza, 2010) and later by others (Bányász et al., 2009; Virág et al., 2009; Bárándi et al., 2010), it was argued that frequency dependent APD changes, including electrical restitution, were determined by the APD at the basic cycle length. The results of the present study partly support this idea, since all of the drugs studied with an APD prolonging effect slowed the restitution curve. Also, longer intrinsic APD was associated with slower restitution in human ventricular muscle. However, mexiletine and nisoldipine shortened basic APD but slowed or did not change the restitution curve. In addition, in the present study, the human ventricular muscle preparations with strong phase 1 repolarization showed slower restitution kinetics with shorter APD at the basic cycle lengths than those that showed no prominent phase 1 repolarization. Rabbit ventricular APD restitution curves showed a declining slope at diastolic intervals longer than 1000 ms. In human ventricular muscle, I_to_ recovers relatively rapidly, with a time constant of 10 ms (our own unpublished observation), but in rabbit the recovery of I_to_ is slower with time constant more than 1 s (Fermini et al., 1992; Sánchez-Chapula et al., 1994). These results suggest that although basic APD has important role in determining restitution slope and kinetics, other transmembrane currents, such as I_Na_ or I_to_, can also play a role in restitution kinetics. This is also in agreement with earlier work in dog Purkinje fibers, where several drugs with I_Na_ and I_Ca-L_ inhibition properties slowed APD restitution (Elharrar et al., 1984). It is also important to note that the basic stimulation frequency, which was 5 times higher in the study of Shattock et al. (2017), can partly explain the differences between their and our present works.

In our study, we used only undiseased donor cardiac ventricular preparations and did not study diseased tissue and atrial muscle. To the best of our knowledge there are no reported *in vitro* drug studies available with the latter preparations. Since APD restitution can be important phenomenon in the mechanism of atrial fibrillation this may be a limitation of our present investigations as such it would be worth to study in the future.

In conclusion, it should be recognized that important species differences exist in the ventricular restitution process including human. Our results indicate that the mechanism of the electrical restitution, at least in undiseased human ventricle, seems complex; and to understand it properly, further studies are needed. Based on our results, in addition to the basic APD, other factors, such as transmembrane ion currents, can influence restitution. The latter raises the possibility that ion channel modifier drugs slowing restitution kinetics may have antiarrhythmic properties by affecting electrical restitution, which may be considered in future drug development projects.

## Data Availability Statement

The datasets generated for this study are available on request to the corresponding author.

## Author Contributions

Conception and design of the experiments: AV, LV. Collection, analysis and interpretation of data: TÁ-L, CL, BB, MB, NN, JT. Drafting the article and revising it critically for intellectual content: IB, NJ, AV, LV.

## Funding

This work was funded by the National Research Development and Innovation Office (NKFIH K-119992 (for AV), K-128851 (for IB), FK-129117 (for NN), and GINOP-2.3.2.-15-2016-00047), the Ministry of Human Capacities Hungary (20391-3/2018/FEKUSTRAT and EFOP-3.6.2-16-2017-00006), the UNKP-19-3-SZTE-5 (New National Excellence Program of the Ministry for Innovation and Technology; for TÁ-L) and János Bolyai Research Scholarship of the Hungarian Academy of Sciences (for NN). The GINOP and EFOP projects are co-financed by the European Union and the European Regional Development Fund.

## Conflict of Interest

The authors declare that the research was conducted in the absence of any commercial or financial relationships that could be construed as a potential conflict of interest.

## Abbreviations

APA, action potential amplitude; APD, action potential duration; APD50, APD measured at 50% of repolarization; APD90, APD measured at 90% of repolarization; ERP, effective refractory period; Vmax, maximum upstroke velocity; RP, resting membrane potential.
